# SynBioTools: a one-stop facility for searching and selecting synthetic biology tools

**DOI:** 10.1186/s12859-023-05281-5

**Published:** 2023-04-17

**Authors:** Pengli Cai, Sheng Liu, Dachuan Zhang, Huadong Xing, Mengying Han, Dongliang Liu, Linlin Gong, Qian-Nan Hu

**Affiliations:** 1 CAS Key Laboratory of Computational Biology, Shanghai Institute of Nutrition and Health, University of Chinese Academy of Sciences, Chinese Academy of Sciences, Shanghai, 200031 China; 2 Ecological Systems Design, Institute of Environmental Engineering, ETH Zurich, 8093 Zurich, Switzerland

**Keywords:** Synthetic biology, Computational tool, Database, Tool retrieval, Tool registry, Table extraction

## Abstract

**Background:**

The rapid development of synthetic biology relies heavily on the use of databases and computational tools, which are also developing rapidly. While many tool registries have been created to facilitate tool retrieval, sharing, and reuse, no relatively comprehensive tool registry or catalog addresses all aspects of synthetic biology.

**Results:**

We constructed SynBioTools, a comprehensive collection of synthetic biology databases, computational tools, and experimental methods, as a one-stop facility for searching and selecting synthetic biology tools. SynBioTools includes databases, computational tools, and methods extracted from reviews via SCIentific Table Extraction, a scientific table-extraction tool that we built. Approximately 57% of the resources that we located and included in SynBioTools are not mentioned in bio.tools, the dominant tool registry. To improve users’ understanding of the tools and to enable them to make better choices, the tools are grouped into nine modules (each with subdivisions) based on their potential biosynthetic applications. Detailed comparisons of similar tools in every classification are included. The URLs, descriptions, source references, and the number of citations of the tools are also integrated into the system.

**Conclusions:**

SynBioTools is freely available at https://synbiotools.lifesynther.com/. It provides end-users and developers with a useful resource of categorized synthetic biology databases, tools, and methods to facilitate tool retrieval and selection.

**Supplementary Information:**

The online version contains supplementary material available at 10.1186/s12859-023-05281-5.

## Background

In synthetic biology research, data processing, computational modeling, and artificial intelligence play important roles in the design and analysis of laboratory experiments [1–3]. For instance, the big data generated by high-throughput sequencing depends on computational data processing. This has promoted the rapid development of databases and computational tools, with large numbers of them being produced in recent decades.

To better manage these resources, various tool registries of different sizes and on different topics have been created, improving convenience for users and developers. These include BioMOBY [4], Bioconductor [5], BioCatalogue [6], SEQanswers (a wiki database of tools for high-throughput sequencing analysis) [7], BioJavaScript (BioJS) for bioinformatics visualization tools [8, 9], the BioContainers Registry [10], OMICtools (a directory of tools for various kinds of omics analyses) [11], Bio-TDS [12], bio.tools [13], JIB.tools 2.0 [14], Expasy [15], and GSARefDB (providing tools for gene set analysis) [16]. Among these registries, bio.tools and BioContainers are currently the largest. The bio.tools registry, based on community-driven curation, lists 25,299 tools [13]. BioContainers stores, creates, and distributes bioinformatics tools, containers, and packages [10]. The various existing tool registries make it easier to find tools during experimental design and analysis or tool development. Nonetheless, there is currently no comprehensive tool registry for synthetic biology. While the existing registries list some useful design and analysis tools for synthetic biology research, some of these tool registries, such as OMICtools [11] and SEQwiki [7] for omics analysis, are no longer available. The Secondary Metabolite Bioinformatics Portal (SMBP) provides the computational tools to facilitate synthetic biology research involving secondary metabolite production [17], but does not offer researchers a one-stop search for finding other tools. Furthermore, comparative information on similar tools is lacking in the large tool registries. From the end user’s perspective, it is often challenging to choose the right tool for each research task from the many similar tools that have been developed over the years.

At the same time, the development of a large number of tools has been accompanied by the publication of reviews describing them. These reviews have efficiently categorized and compared similar tools or databases for different topics or categories, addressing some of the problems related to the tool registries mentioned. These reviews are, therefore, extremely valuable resources for tool users and developers. Nonetheless, information about the tools is scattered among different reviews, and the information provided by these reviews cannot be explored interactively, as is possible with tool registries.

To address these issues, we constructed SynBioTools, a registry dedicated to synthetic biology tools, with relevant databases, computational tools, and methods. Some relevant experimental methods and tools, such as DNA assembly tools, were integrated for coherence and convenience. These resources were collected from review articles dealing with tools and databases in synthetic biology. To better extract information from reviews, we built SCIentific Table Extraction (SCITE), a tool for extracting tabular data from articles. We extracted information on tool classification, features, and comparisons, and reorganized it into biosynthetic tool categories. SynBioTools combines the advantages of the reviews’ categorical summaries and human–computer interactions via a web-server database. We further integrated other tool-related information to help users to select the appropriate tools to match their needs.

## Methods

### Data acquisition

We retrieved references for bioinformatics tools from bio.tools, which provides a comprehensive registry of tools and databases. Additionally, the Semantic Scholar Open Research Corpus (S2ORC) dataset (https://allenai.org/data/s2orc) and PubMed data (https://pubmed.ncbi.nlm.nih.gov/download/) were downloaded as data sources for all literature. The S2ORC and PubMed data were used to obtain citations and review labels. To obtain reviews describing bioinformatics tools, we extracted citations for all tools from the S2ORC dataset, filtered them for review articles, and then selected reviews citing more than 100 tools that were published between 2010 and 2022. Synthetic biology-related reviews were chosen manually for further tool information extraction. Finally, 37 review articles were used for tool extraction. We used our custom-developed tool, SCITE, to extract information from the tables in the reviews. Based on their characteristics and biosynthetic process application [18], we manually grouped the tools and databases into nine modules: compounds, biocomponents, protein, pathway, gene-editing, metabolic modeling, omics, strains, and others.

### Tabular information extraction

To extract information from the tables in the reviews, we developed a literature-table-extraction tool, SCITE, based on the optical character recognition (OCR) toolkit PaddleOCR (https://github.com/PaddlePaddle/PaddleOCR) and the R package tidypmc (https://github.com/ropensci/tidypmc). SCITE implements two methods to extract tables from articles. For general articles in PDF format, we built a table extraction tool based on an OCR strategy (Additional file 1). This tool first converts the pages of a PDF document into image format, then identifies and extracts the table information from the images based on PaddleOCR, which is an ultra-light deep learning OCR model. For papers from PubMed Central, we obtained tables by parsing the full-text XML file directly using tidypmc (Additional file 2). We further deployed SCITE as an API using FastAPI and Celery. Finally, the tabular information from review articles was automatically extracted using SCITE.

### Data curation and integration

Data management and integration included table extraction, manual curation, data supplement, and data integration. As most of the tables were formatted differently between papers and the automatically extracted data were not 100% reliable, manual curation was performed after table extraction by SCITE. During the curation process, we corrected some mistakes and formatted each row to one tool. Based on the reference columns in the review tables, we obtained and supplemented direct references for each tool using either programming or manual means. They were subsequently used to obtain information on reference-related common fields. The data integrated into SynBioTools is divided into common and unique fields. Common fields, such as name, module, citation, and other information common to all tools, are displayed on the SynBioTools Browse page, while unique field information from the review table is displayed on the tool Details page.

### System design and implementation

The SynBioTools web server, deployed in the Ubuntu 18.04.2 environment, uses the Python Web framework FastAPI 0.73.0 and the front-end framework Bootstrap 5.2. The project data are stored in the NoSQL database MongoDB 5.0.4. We used the JavaScript libraries Echarts 5.3.3 and Tabulator 5.4.2 for graph and table rendering, respectively. We further developed various search methods using Elasticsearch 7.16.2. SynBioTools is freely available at https://synbiotools.lifesynther.com. Users can access it in Google Chrome or Safari for the best experience.

## Results

### SynBioTools summary

**Fig. 1:**
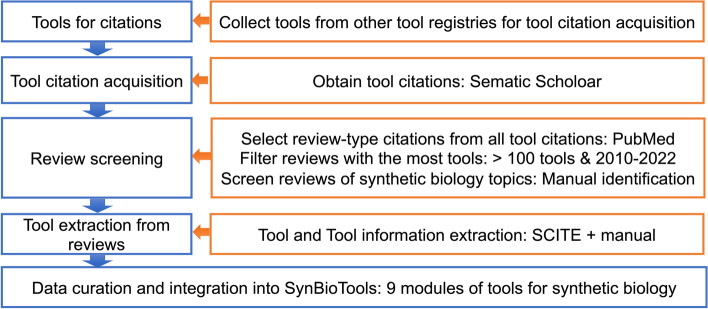
Schematic of the process of constructing SynBioTools. Tools and tool information were extracted via the following steps: collect tools from other tool registries to obtain tool citations; select only citations for reviews; filter for reviews published from 2010 to 2022 with more than 100 tool citations; manually select review articles on synthetic biology. After data curation and integrating information such as the tool’s URL, description, reference, and the number of citations, the tools were grouped into nine modules, i.e., compounds, biocomponents, protein, pathway, gene-editing, metabolic modeling, omics, strains, and others, based on their potential biosynthetic applications

SynBioTools is a one-stop solution for searching and selecting synthetic biology tools. Here, synthetic biology tools refer to the tools, methods, and databases used for synthetic biology research. All the tools in SynBioTools were extracted from review articles [1, 18–53] (Additional file 3) via SCITE, our custom-built article-table-extraction tool. The method and process of the construction of SynBioTools are summarized in Fig. 1. Based on the tool characteristics and potential biosynthesis application, we manually grouped them into nine modules (compounds, biocomponents, protein, pathway, gene-editing, metabolic modeling, omics, strains, and others), related to compound selection, pathway mining and design, element selection, protein selection and design, gene editing, metabolic network modeling, omics analysis, and strain modification, respectively. Additional parameters were integrated, including tool descriptions, source references, URLs linking to the tools, and hints toward tool availability on the Browse page. The probability that a tool’s web server is accessible is positively correlated with the number of citations of that tool [54], and article citation counts are used to estimate tool popularity [16]. Therefore, for each tool, we provided the total numbers of all citations, review citations, citations used for tool development, and citations reflecting the experimental application of the tool (i.e., not including the previously mentioned review and tool-development articles). This grouping and the parameters included will improve users’ understanding and selection of tools.

**Fig. 2:**
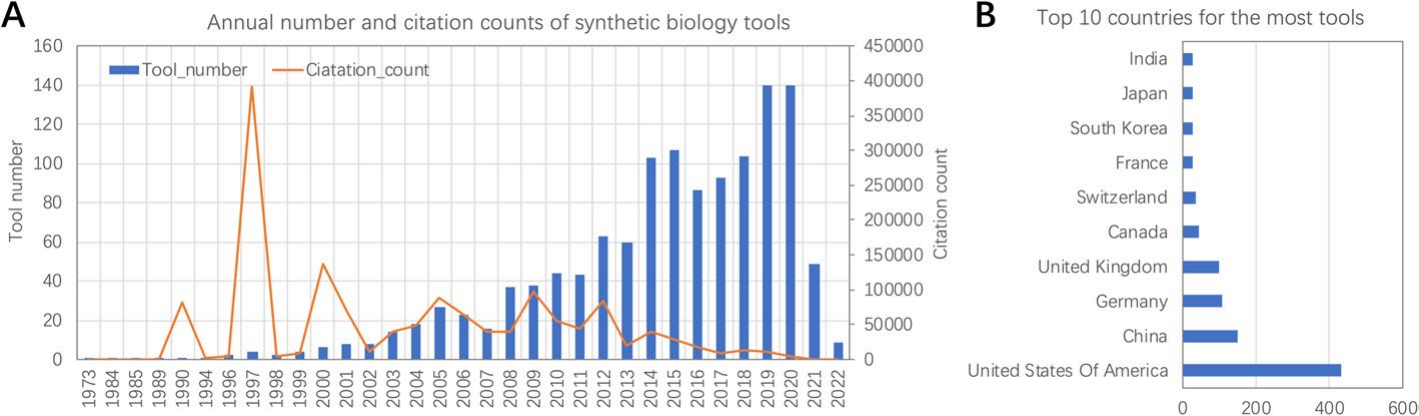
Summary of the data in SynBioTools. **A** Annual numbers of tools published and tool citations in synthetic biology. The citation count refers to the annual number of citations of all tools. Most of the tools and databases included in SynBioTools were developed within the last 20 years. The tools and databases with the most citations (and year of origin) are BLAST (1990), KEGG (1997), GO (2000), STRING (2000), NCBI (2000), MAFFT (2005), Reactome (2005), PRIDE (2005), Bowtie (2009), Fastree (2009), and Bowtie 2 (2012), with the years corresponding to five highest citation-count peaks. **B** Top 10 countries contributing the most tools to SynBioTools

**Fig. 3:**
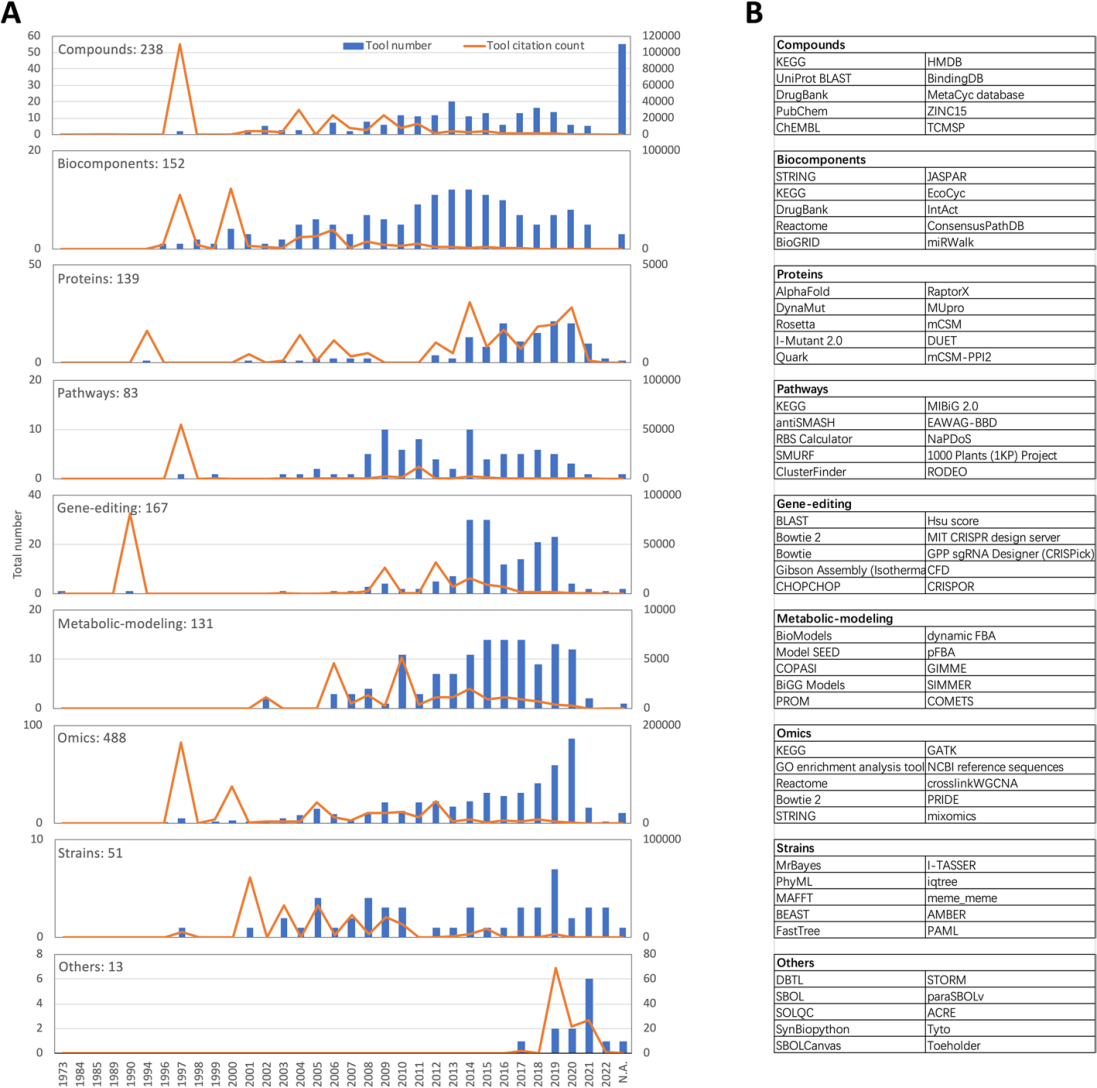
Tools in each SynBioTools module. **A** Annual tool number and annual citation count for each of the nine modules. The number of tools in each module is listed next to the module name. **B** Top 10 most-cited tools in each of the nine modules

Most of the tools and databases included in SynBioTools were developed within the last 20 years (Fig. 2A). In the past 10 years, the number of tools has increased rapidly, while the number of citations has declined. Familiar and frequently used tools, such as BLAST, KEGG, GO, STRING, NCBI, MAFFT, Reactome, PRIDE, Fastree, and Bowtie, have numerous citations (Fig. 2A). The top three countries developing the tools or databases listed in SynBioTools are the United States of America, China, and Germany (Fig. 2B). Based on the annual numbers of tools and citations for each module, most of the tools in most of the modules were developed within the past 20 years. Most of the tools in the protein, gene editing, metabolic modeling, and omics modules were developed within the past 10 years (Fig. 3A). SynBioTools lists 1321 de-duplicated tools and 1462 tool records, because some comprehensive tools or databases, such as KEGG, were grouped into more than one module (Fig. 3B). The top 10 tools in terms of citation counts are BLAST, MrBayes, KEGG, GO enrichment analysis tool, PhyML, Bowtie 2, STRING, UniProt BLAST, MAFFT, and BEAST. According to the published sources for each tool, the top 10 databases and tools that are continually updated include KEGG, UniProt BLAST, CTD, NCBI reference sequences, PubChem, EcoCyc, RegulonDB, Reactome, the MetaCyc database, and STRING. SynBioTools shares 564 tools with bio.tools, which is the primary tool registry; of the 757 not shared with bio.tools, 62 are for laboratory experiments, providing cloning strategies and DNA-assembly methods that are critical in synthetic biology. Including these tools provides a one-stop search solution for synthetic biology tools.

### User interface

**Fig. 4:**
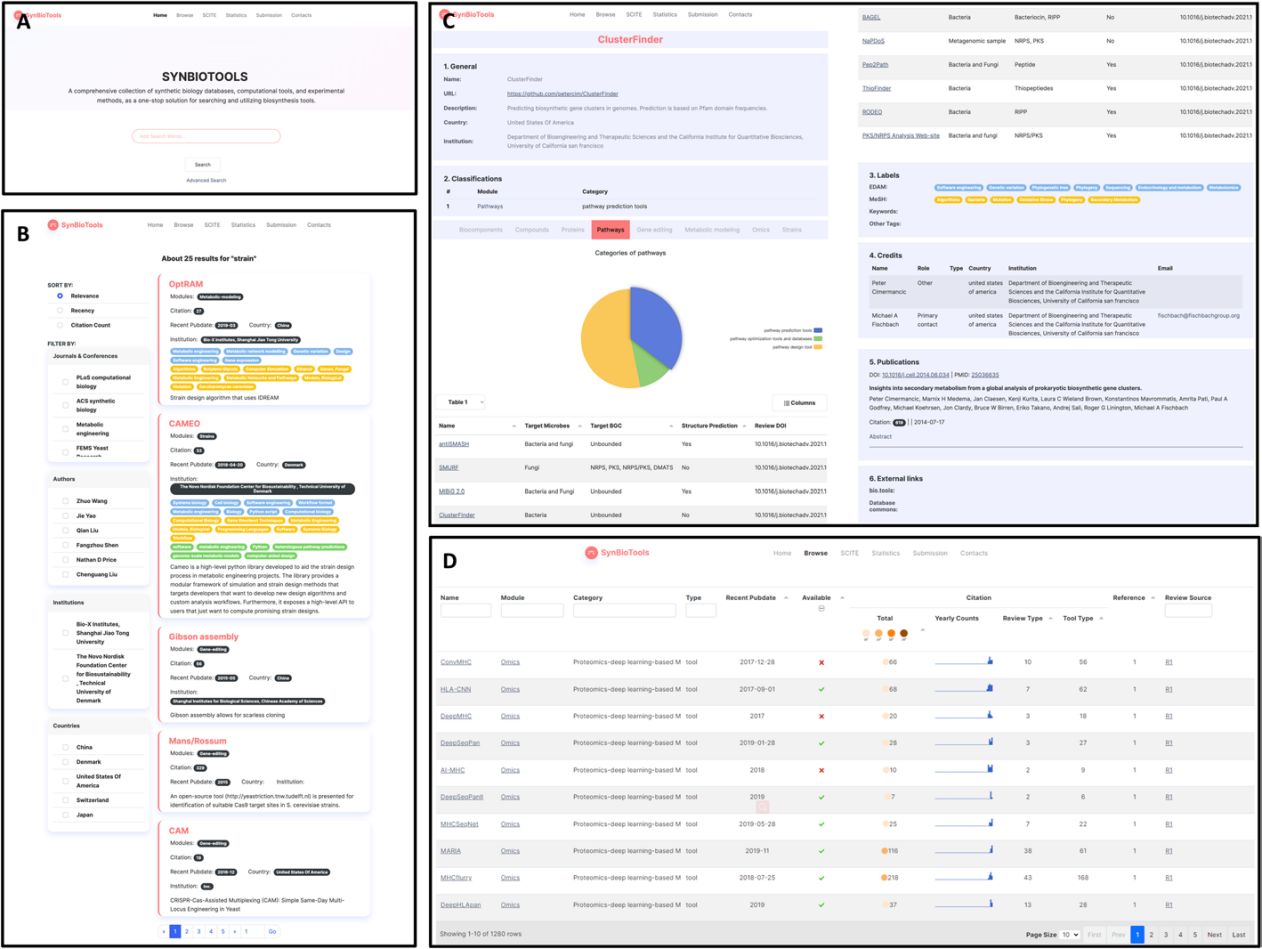
SynBioTools content and web interface. **A** Home Search page, including simple and advanced search. **B** Search Results page. The results can be sorted by relevance, recency, or citation count, and filtered by journals, authors, institutions, and countries. **C** Tool Details page, including general information, classification, labels, credits, publications, and external links. **D** Browse page, showing the tool’s name, module, category, data type, homepage availability, publication date, citation, tool reference, and review source

On the Home Search page, SynBioTools offers two retrieval methods: simple and advanced search (Fig. 4A). In the simple search, possible tools will be displayed while the search term is being typed. For an advanced search, the search term can be the tool name, module, keyword, EDAM term, MeSH term, author, country, institution, or any other term, and search terms can be combined. On the Search Results page, the retrieved tools are shown on the right, with the sorting methods and filtering criteria on the left (Fig. 4B). The tools can be sorted by relevance, recency, and citation count, and filtered by journals, conferences, authors, institutions, and countries. Clicking on the tool name in the search result will load the Tool Details page (Fig. 4C), which includes general information, classifications, labels, credits, publications, and external links, lists other tools in the same category, and provides comparisons with these tools.

The Browse page displays the tool name, module, category, type, publication date, homepage availability, citation, source reference, and review source, allowing tool information retrieval and sorting (Fig. 4D). The Tool Details page can also be accessed by clicking on the tool name on the Browse page.

Our article-table-extraction tool, SCITE, has been integrated into SynBioTools as an online server application. SCITE provides two ways to extract tabular data from scientific papers, and users can choose the mode based on the file type. If a PDF file of an article is uploaded, SCITE will automatically convert the uploaded file into pictures, and identify tables via artificial intelligence. If the user provides an article’s PMCID from PubMed Central, SCITE will extract the table information by parsing the full-text XML document, providing more accurate table retrieval. SCITE can be accessed freely at https://synbiotools.lifesynther.com/scite.html.

## Discussion

Synthetic biology research involves the utilization of many databases and computational tools. We constructed SynBioTools, comprehensively listing categorized synthetic biology tools, to make it easier to search and select biosynthetic tools and conduct synthetic biology research. SynBioTools lists computational tools, databases, and methods grouped into nine modules based on their potential biosynthetic applications. Unlike existing registries, SynBioTools lists tools, databases, and methods related to most biosynthesis processes in order to facilitate tool discovery, sharing, and reutilization across the field of synthetic biology. SynBioTools also includes experimental laboratory methods, such as DNA assembly and cloning strategies, to allow researchers to locate and retrieve all methods in one place. Approximately 57% of the tools listed in SynBioTools are not found in the most comprehensive tool registry, bio.tools. Although OMICtools lists a larger number of omics analysis tools and has a good classification system, it is currently not available [14]. Additionally, while SMBP provides computational tools for secondary metabolite production, it does not offer researchers a one-stop search facility for other tools [17].

As well as enabling tool retrieval, SynBioTools provides a comprehensive overview of synthetic biology tools and includes a wealth of tools and database resources for constructing workflows and large comprehensive databases. It reveals that the number of synthetic biology tools has grown rapidly in the past 20 years, especially in the fields of omics and gene editing; this growth is closely related to the emergence and rapid development of sequencing and CRISPR/Cas technologies. Omics and gene editing are driving rapid technological developments in synthetic biology [37]. Genome editing, via programmable nucleases, is revolutionizing the life sciences and medicine; currently available CRISPR/Cas-related tools facilitate convenient and reliable genome-editing experiments at every step, from designing guide RNA to analyzing gene editing outcomes [31]. In recent years, the enormous progress in developing protein design tools has promoted rapid development in the field of protein design. Protein design is no longer restricted to fundamentals and the analysis of protein folding. Our ability to generate and manipulate synthetic proteins has advanced to the point where they provide realistic alternatives to the functions of natural proteins for both in vitro and intracellular applications. Furthermore, computer-based protein design is becoming increasingly accepted by non-specialists [55]. The collation and classification that SynBioTools provides are conducive to the integration and construction of larger and more comprehensive databases, such as COCONUT, an aggregated open-source dataset of known and predicted natural products [56], as well as integration and interoperability between databases [57]. Workflows can integrate multiple tools to handle analyses that are too complex to be addressed using a single tool [58]. SynBioTools is conducive to the construction of workflows for complex, multi-task data analyses, integrating tools for every step, from chemical selection to pathway design, enzyme selection, gene editing, and omics analysis.

When constructing SynBioTools, we encountered various difficulties, including those related to tabular information extraction and data de-duplication. Data acquisition was a critical step in constructing our tool registry. The current commonly used PDF table batch-extraction tools for extracting structured data from the literature are Tabula (https://github.com/tabulapdf/tabula) and Camelot (https://github.com/camelot-dev/camelot), which have been used for table extraction [59, 60]. However, for some PDF documents, these tools do not perform very well. Therefore, to improve performance and generality, we developed SCITE, which can better extract tabular data from reviews and other types of scientific papers. Further, SynBioTools provides a new strategy for data extraction: find reviews that cite the tool from the identified tools, filter the reviews for the topics of interest, then acquiring additional tools and information from the screened reviews. This makes it possible to rapidly locate topic-specific tools and tool information.

Duplicate removal and tool updates presented difficulties in terms of data curation during our construction of SynBioTools. For example, the same tool may be referred to in different source papers, requiring the merging of records. However, tool disambiguation is difficult because tools do not have a unique identification number. Therefore, we identified unique tools based on the tool name, reference, link, and other factors. Further, some tool updates are described in published articles, while others are provided as ongoing updates. If each tool could be assigned a unique ID number through a system or platform upon tool release, and all updates are linked to the same ID, this would provide a potential solution. However, this would depend on consensus among all tool publishers and publication journals, as well as ID registration and maintenance platforms.

All of the tools in SynBioTools were extracted from reviews. However, due to the publication lag for review articles, the list includes little to no tools that have appeared within the past two years. To address this, we added a small number of synthetic biology tools that are not derived from the review literature. Additionally, we provide a channel for users to manually submit tool information. In the future, given the constant publication of synthetic biology reviews, we will regularly update the data in SynBioTools. This includes updating changes to existing tools and adding new tools to SynBioTools. Concretely, we will perform the data process steps shown in Fig. 1. The only difference is to remove reviews that have been previously processed. In addition, due to the lagging nature of the review literature, we will periodically add synthetic biology tools that are not derived from the review to provide basic tool search, although these tools lack information like detailed comparisons of similar tools extracted from reviews. At the same time, new natural language processing techniques will be applied to optimize the entire data processing pipeline to minimize the reliance on expert curation. SynBioTools focuses on synthetic biology, rather than attempting to address all aspects of computational biology. Nevertheless, it presents a useful catalog of synthetic biology tools for researchers and tool developers.

## Conclusions

We constructed SynBioTools, which includes computational tools, databases, and methods, to improve the ease of locating tools used in synthetic biology. SynBioTools combines the advantages of data collation and comparison of review articles with the ease of interaction of databases. It extracts biosynthesis-related tools from published reviews of synthetic biology tools, classifies them according to their characteristics and potential biosynthetic applications, and integrates extra information, such as tool URLs, source references, and the number of citations, to assist users and developers in tool retrieval and selection. SynBioTools provides researchers with an efficient, one-stop search and selection facility for finding synthetic biology tools, as well as a source of tools for further workflow construction.

## Supplementary Information

**Additional file 1**. The code zip file on extracting tabular information from papers in PDF format based on OCR.

**Additional file 2**. The code zip file on extracting tabular information from papers by paring the full-text XML file.

**Additional file 3**. The list of reviews used for the tool and tool information extraction.

## Data Availability

The datasets used and analyzed during the current study are available from the corresponding author upon reasonable request.

## Abbreviations

BioJS: BioJavaScript
SMBP: Secondary Metabolite Bioinformatics Portal
SCITE: SCIentific Table Extraction
S2ORC: Semantic Scholar Open Research Corpus
OCR: Optical Character Recognition
