# Supplementary material for: SynBioTools: a one-stop facility for searching and selecting synthetic biology tools
Source: BMC Bioinformatics. 2023 Apr 17;24:152. doi: 10.1186/s12859-023-05281-5 (PMC10111727; doi:10.1186/s12859-023-05281-5)
Supplement: Supplementary file 3 — Additional file 3. The list of reviews used for the tool and tool information extraction. [file 12859_2023_5281_MOESM3_ESM.docx]

The list of reviews used for the tool and tool information extraction:

1. Zielinski DC, Patel A, Palsson BO: **The Expanding Computational Toolbox for Engineering Microbial Phenotypes at the Genome Scale**. *Microorganisms* 2020, **8**(12).

2. Majewska M, Wysokińska H, Kuźma Ł, Szymczyk P: **Eukaryotic and prokaryotic promoter databases as valuable tools in exploring the regulation of gene transcription: a comprehensive overview**. *Gene* 2018, **644**:38-48.

3. Misra BB, Langefeld CD, Olivier M, Cox LA: **Integrated Omics: Tools, Advances, and Future Approaches**. *J Mol Endocrinol* 2018.

4. Gu C, Kim GB, Kim WJ, Kim HU, Lee SY: **Current status and applications of genome-scale metabolic models**. *Genome Biol* 2019, **20**(1):121.

5. Chen C, Hou J, Tanner JJ, Cheng J: **Bioinformatics Methods for Mass Spectrometry-Based Proteomics Data Analysis**. *Int J Mol Sci* 2020, **21**(8).

6. Dhingra S, Sowdhamini R, Cadet F, Offmann B: **A glance into the evolution of template-free protein structure prediction methodologies**. *Biochimie* 2020, **175**:85-92.

7. Ejigu GF, Jung J: **Review on the Computational Genome Annotation of Sequences Obtained by Next-Generation Sequencing**. *Biology (Basel)* 2020, **9**(9).

8. Guala D, Ogris C, Muller N, Sonnhammer ELL: **Genome-wide functional association networks: background, data & state-of-the-art resources**. *Brief Bioinform* 2020, **21**(4):1224-1237.

9. Hanna RE, Doench JG: **Design and analysis of CRISPR-Cas experiments**. *Nat Biotechnol* 2020, **38**(7):813-823.

10. Kapli P, Yang Z, Telford MJ: **Phylogenetic tree building in the genomic age**. *Nat Rev Genet* 2020, **21**(7):428-444.

11. Makrodimitris S, van Ham R, Reinders MJT: **Automatic Gene Function Prediction in the 2020's**. *Genes (Basel)* 2020, **11**(11).

12. McCarty NS, Graham AE, Studena L, Ledesma-Amaro R: **Multiplexed CRISPR technologies for gene editing and transcriptional regulation**. *Nat Commun* 2020, **11**(1):1281.

13. Ren H, Shi C, Zhao H: **Computational Tools for Discovering and Engineering Natural Product Biosynthetic Pathways**. *iScience* 2020, **23**(1):100795.

14. Sledzinski P, Nowaczyk M, Olejniczak M: **Computational Tools and Resources Supporting CRISPR-Cas Experiments**. *Cells* 2020, **9**(5).

15. Sorokina M, Steinbeck C: **Review on natural products databases: where to find data in 2020**. *J Cheminform* 2020, **12**(1):20.

16. Wen B, Zeng WF, Liao Y, Shi Z, Savage SR, Jiang W, Zhang B: **Deep Learning in Proteomics**. *Proteomics* 2020, **20**(21-22):e1900335.

17. Alam K, Hao J, Zhang Y, Li A: **Synthetic biology-inspired strategies and tools for engineering of microbial natural product biosynthetic pathways**. *Biotechnol Adv* 2021, **49**:107759.

18. Ayres LB, Gomez FJV, Linton JR, Silva MF, Garcia CD: **Taking the leap between analytical chemistry and artificial intelligence: A tutorial review**. *Anal Chim Acta* 2021, **1161**:338403.

19. Baltoumas FA, Zafeiropoulou S, Karatzas E, Koutrouli M, Thanati F, Voutsadaki K, Gkonta M, Hotova J, Kasionis I, Hatzis P *et al*: **Biomolecule and Bioentity Interaction Databases in Systems Biology: A Comprehensive Review**. *Biomolecules* 2021, **11**(8).

20. Bao XR, Pan Y, Lee CM, Davis TH, Bao G: **Tools for experimental and computational analyses of off-target editing by programmable nucleases**. *Nat Protoc* 2021, **16**(1):10-26.

21. Bin Hafeez A, Jiang X, Bergen PJ, Zhu Y: **Antimicrobial Peptides: An Update on Classifications and Databases**. *Int J Mol Sci* 2021, **22**(21).

22. Chung CH, Lin DW, Eames A, Chandrasekaran S: **Next-Generation Genome-Scale Metabolic Modeling through Integration of Regulatory Mechanisms**. *Metabolites* 2021, **11**(9).

23. Jendoubi T: **Approaches to Integrating Metabolomics and Multi-Omics Data: A Primer**. *Metabolites* 2021, **11**(3).

24. Luo J, Wei Y, Lyu M, Wu Z, Liu X, Luo H, Yan C: **A comprehensive review of scaffolding methods in genome assembly**. *Brief Bioinform* 2021, **22**(5).

25. Marabotti A, Scafuri B, Facchiano A: **Predicting the stability of mutant proteins by computational approaches: an overview**. *Brief Bioinform* 2021, **22**(3).

26. Misra BB: **New software tools, databases, and resources in metabolomics: updates from 2020**. *Metabolomics* 2021, **17**(5):49.

27. Pakhrin SC, Shrestha B, Adhikari B, Kc DB: **Deep Learning-Based Advances in Protein Structure Prediction**. *Int J Mol Sci* 2021, **22**(11).

28. Pereira JM, Vieira M, Santos SM: **Step-by-step design of proteins for small molecule interaction: A review on recent milestones**. *Protein Sci* 2021, **30**(8):1502-1520.

29. Santiago-Rodriguez TM, Hollister EB: **Multi 'omic data integration: A review of concepts, considerations, and approaches**. *Semin Perinatol* 2021, **45**(6):151456.

30. Sequeiros-Borja CE, Surpeta B, Brezovsky J: **Recent advances in user-friendly computational tools to engineer protein function**. *Brief Bioinform* 2021, **22**(3).

31. Suthers PF, Foster CJ, Sarkar D, Wang L, Maranas CD: **Recent advances in constraint and machine learning-based metabolic modeling by leveraging stoichiometric balances, thermodynamic feasibility and kinetic law formalisms**. *Metab Eng* 2021, **63**:13-33.

32. Worheide MA, Krumsiek J, Kastenmuller G, Arnold M: **Multi-omics integration in biomedical research - A metabolomics-centric review**. *Anal Chim Acta* 2021, **1141**:144-162.

33. Wu M, Yi H, Ma S: **Vertical integration methods for gene expression data analysis**. *Brief Bioinform* 2021, **22**(3).

34. Young R, Haines M, Storch M, Freemont PS: **Combinatorial metabolic pathway assembly approaches and toolkits for modular assembly**. *Metab Eng* 2021, **63**:81-101.

35. Zou Y, Zhu Y, Li Y, Wu FX, Wang J: **Parallel computing for genome sequence processing**. *Brief Bioinform* 2021, **22**(5).

36. Luo L, Yang J, Wang C, Wu J, Li Y, Zhang X, Li H, Zhang H, Zhou Y, Lu A *et al*: **Natural products for infectious microbes and diseases: an overview of sources, compounds, and chemical diversities**. *Sci China Life Sci* 2022, **65**(6):1123-1145.

37. Otero-Muras I, Carbonell P: **Automated engineering of synthetic metabolic pathways for efficient biomanufacturing**. *Metab Eng* 2021, **63**:61-80.
